# Supplementary material for: Disease activity trajectories for early and established rheumatoid arthritis: Real-world data from a rheumatoid arthritis cohort
Source: PLoS One. 2022 Sep 7;17(9):e0274264. doi: 10.1371/journal.pone.0274264 (PMC9451079; doi:10.1371/journal.pone.0274264)
Supplement: S1 Appendix — (DOCX) [file pone.0274264.s001.docx]

**Table A1 Suppl.** **Fit statistics for the disease course (DAS28-ESR) trajectory model**

**in patients with early RA**

| N=660 |  |  |  |  |
| --- | --- | --- | --- | --- |
| Group size (%) |  |  |  |  |
| 1 | 100 | 66.1 | **19.1** | 17.8 |
| 2 | - | 33.9 | **54.0** | 53.5 |
| 3 | - | - | **26.9** | 3.5 |
| 4 | - | - | **-** | 25.3 |
| BIC | -5965.35 | -5546.78 | **-5455.78** | -5458.06 |

Note: Fit statistics and model selection was based on “log Bayes factor which is calculated as:

2× [((BIC for current model)- (BIC for previous model)]. If log Bayes factor is a negative value,

we stop and select the previous model. If log Bayes factor is a positive value,

we move to the next model by adding another group (5, 7)

**Table A2 Suppl. The mean (95% CI) CDAI values at each time point for trajectory classes in patients with early RA**

| **N=660** | **Group 1**  **MD-REM** | **Group 2**  **MD-LDA** | **Group 3**  **MD-MD** | **Group 4**  **HD-LDA** | **Group 5**  **HD-HD** | **Group 6**  **VHD-MD** | **Group 7**  **HD-HD** |
| --- | --- | --- | --- | --- | --- | --- | --- |
| **Group percent** | **1.9%** | **44.4%** | **22.1%** | **19.5%** | **4.9%** | **4.3%** | **2.9%** |
| Baseline | 17.5 (6.18-28.8) | 14.0 (11.8-15.3) | 20.1 (17.7-22.6) | 34.3 (31.9-36.6) | 30.4 (23.8-37.0) | 50.7 (47.1-54.4) | 39.4 (35.3-43.5) |
| 6 months | 0.83 (0.0-5.84) | 8.84 (7.38-10.3) | 18.5 (16.2-20.9) | 16.7 (15.1-18.3) | 26.1 (20.5-31.6) | 37.4 (34.8-40.0) | 41.7 (38.0-45.5) |
| 12 months | 0.01 (0.0-2.09) | 6.29 (4.81-7.78) | 16.9 (14.4-19.5) | 7.27 (5.43-9.11) | 24.6 (19.3-29.9) | 26.6 (23.6-29.5) | 41.3 (37.3-45.3) |
| 18 months | 0.002 (0.0-0.96) | 5.43 (4.10-6.77) | 15.3 (12.3-18.4) | 5.07 (3.79-6.35) | 26.0 (20.7-31.3) | 18.3 (15.4-21.3) | 38.2 (35.0-41.4) |
| 24 months | 0.047 (0.0-8.27) | 5.99 (4.48-7.50) | 13.8 (10.0-17.5) | 8.32 (6.28-10.4) | 30.3 (23.4-37.1) | 12.8 (8.51-17.0) | 32.4 (28.3-36.4) |

**Table A3 Suppl. Cross tabulation between DAS28 and CDAI subgroups in patients with early RA**

| **N=660** | **DAS28 subgroups** | | |
| --- | --- | --- | --- |
| **CDAI subgroups** | **Group 1**  **LDA-REM**  **N=110** | **Group 2**  **MD-REM**  **N=371** | **Group 3**  **HD-MD**  **N=179** |
| **Group-1 (MD-REM)**  **N=12** | 7 (58.3) | 5 (41.7) | 0 |
| **Group-2 (MD-LDA)**  **N=301** | 97 (32.2) | 187 (62.1) | 17 (5.7) |
| **Group-3 (MD-MD)**  **N=145** | 4 (2.8) | 72 (49.7) | 69 (47.6) |
| **Group-4 (HD-LDA)**  **N=124** | 2 (1.6) | 103 (83.1) | 19 (15.3) |
| **Group-5 (HD-HD)**  **N=31** | 0 | 1 (3.2) | 30 (96.8) |
| **Group-6 (VHD-MD)**  **N=27** | 0 | 3 (11.1) | 24 (88.9) |
| **Group-7 (HD-HD)**  **N=20** | 0 | 0 | 20 (100) |

**Table A4 Suppl. The mean (95% CI) DAS28-ESR values at each time point for trajectory classes in patients with established RA**

| **N=1260** | **Group 1**  **REM-REM** | **Group 2**  **HD-REM** | **Group 3**  **LDA-LDA** | **Group 4**  **MD-MD** | **Group 5**  **HD-LDA** | **Group 6**  **HD-MD** | **Group 7**  **HD-HD** |
| --- | --- | --- | --- | --- | --- | --- | --- |
| **Group percent** | **18.3%** | **14.2%** | **29.8%** | **18.1%** | **10.3%** | **3.3%** | **6.1%** |
| Baseline | 2.28 (2.12-2.43) | 5.04 (4.81-5.27) | 3.25 (3.12-3.38) | 4.44 (4.28-4.61) | 5.56 (5.31-5.80) | 6.91 (6.29-7.53) | 5.86 (5.63-6.09) |
| 6 months | 1.78 (1.65-1.90) | 3.38 (3.22-3.54) | 3.13 (3.03-3.24) | 4.25 (4.29-4.56) | 4.86 (4.69-5.04) | 6.23 (8.82-6.65) | 5.87 (5.69-6.05) |
| 12 months | 1.52 (1.37-1.66) | 2.38 (2.19-2.57) | 3.02 (2.92-3.11) | 4.41 (4.28-4.53) | 4.17 (4.02-4.33) | 5.56 (5.30-5.83) | 5.88 (5.70-6.07) |
| 18 months | 1.49 (1.36-1.63) | 2.04 (1.85-2.23) | 2.90 (2.79-3.01) | 4.39 (4.24-4.54) | 3.48 (3.28-3.68) | 4.89 (4.64-5.14) | 5.89 (5.68-6.12) |
| 24 months | 1.70 (1.54-1.86) | 2.36 (2.15-2.56) | 2.78 (2.65-2.92) | 4.37 (4.17-4.57) | 2.79 (2.51-3.07) | 4.21 (3.83-4.60) | 5.90 (5.59-6.21) |

**Table A5 Suppl. The mean (95% CI) CDAI values at each time point for trajectory classes in patients with established RA**

| **N=1260** | **Group 1**  **LDA-LDA** | **Group 2**  **MD-MD** | **Group 3**  **HD-HD** | **Group 4**  **HD-LDA** | **Group 5**  **HD-HD** | **Group 6**  **VHD-LDA** | **Group 7**  **VHD-VHD** |
| --- | --- | --- | --- | --- | --- | --- | --- |
| **Group percent** | **37.1%** | **31.1%** | **8.9%** | **10.9%** | **4.4%** | **7.0%** | **0.6%** |
| Baseline | 9.21 (8.03-10.4) | 15.9 (14.6-17.2) | 23.3 (21.3-25.3) | 33.6 (31.2-36.0) | 36.5 (34.2-38.8) | 40.8 (38.4-43.3) | 52.5 (47.1-57.9) |
| 6 months | 6.29 (5.50-7.07) | 14.6 (13.5-15.8) | 23.5 (21.9-25.1) | 16.3 (14.7-18.0) | 36.9 (35.1-38.8) | 31.3 (29.2-33.4) | 54.8 (51.0-58.5) |
| 12 months | 4.81 (4.13-5.50) | 13.4 (12.3-14.5) | 23.7 (22.2-25.3) | 6.92 (4.75-9.09) | 36.4 (34.4-38.3) | 22.8 (20.6-25.0) | 53.8 (49.7-57.8) |
| 18 months | 4.43 (3.85-5.02) | 12.2 (10.9-13.4) | 23.9 (22.1-25.8) | 4.61 (3.09-6.12) | 34.8 (33.2-36.5) | 15.5 (13.7-17.4) | 49.5 (48.1-53.0) |
| 24 months | 5.03 (4.21-5.85) | 11.0 (9.43-12.6) | 24.1 (21.7-26.6) | 7.45 (5.38-9.51) | 32.3 (30.-34.6) | 9.54 (6.90-12.2) | 42.0 (37.0-47.1) |

**Table A6 Suppl. Cross tabulation between DAS28 and CDAI subgroups in patients with established RA**

| **N=1260** | **DAS28 subgroups** | | | | | | |
| --- | --- | --- | --- | --- | --- | --- | --- |
| **CDAI subgroups** | **Group 1**  **REM-REM**  **N=231** | **Group 2**  **HD-REM**  **N=175** | **Group 3**  **LDA-LDA**  **N=384** | **Group 4**  **MD-MD**  **N= 235** | **Group 5**  **HD-LDA**  **N=121** | **Group 6**  **HD-MD**  **N=38** | **Group 7**  **HD-HD**  **N=76** |
| **Group-1 (LDA-LDA)**  **N=478** | 205 (42.9) | 59 (12.3) | 199 (41.6) | 13 (2.7) | 2 (0.42) | 0 | 0 |
| **Group-2 (MD-MD)**  **N=397** | 19 (4.8) | 38 (9.6) | 163 (41.1) | 132 (33.3) | 40 (10.1) | 2 (0.50) | 3 (0.76) |
| **Group-3 (HD-HD)**  **N=104** | 0 | 2 (1.9) | 11 (10.6) | 58 (55.8) | 5 (4.8) | 1 (0.96) | 27 (26.0) |
| **Group-4 (HD-LDA)**  **N=130** | 7 (5.4) | 72 (55.4) | 6 (4.6) | 15 (11.5) | 29 (22.3) | 0 | 1 (0.77) |
| **Group-5 (HD-HD)**  **N=56** | 0 | 0 | 0 | 12 (21.4) | 1 (1.8) | 9 (16.1) | 34 (60.7) |
| **Group-6 (VHD-LDA)**  **N=87** | 0 | 4 (4.6) | 5 (5.8) | 5 (5.8) | 44 (50.6) | 24 (27.6) | 5 (5.8) |
| **Group-7 (VHD-VHD)**  **N=8** | 0 | 0 | 0 | 0 | 0 | 2 (25.0) | 6 (75.0) |

**Table A7 Suppl. The association between DAS28 subgroups and HAQ-DI and pain improvement in patients with early RA**

|  | **Group 1**  **LDA-REM**  **N=110** | **Group 2**  **MD-REM**  **N=371** | **Group 3**  **HD-MD**  **N=179** |
| --- | --- | --- | --- |
| **Change in HAQ-DI, Mean (SD)** |  |  |  |
| - At 12 months | -0.20 (0.44) | -0.40 (0.64) | -0.27 (0.66) |
| - At 24 months | -0.20 (0.51) | -0.42 (0.73) | -0.31 (0.65) |
| **Change in Pain, Mean (SD)** |  |  |  |
| - At 12 months | -0.32 (0.71) | -0.47 (0.93) | -0.24 (0.95) |
| - At 24 months | -0.37 (0.83) | -0.53 (0.96) | -0.35 (0.87) |

**Table A8 Suppl. The estimate and standard error for DAS28-ESR from the final latent growth curve models in patients with early RA**

Maximum Likelihood Estimates

Model: Censored Normal (CNORM)

Standard T for H0:

Group Parameter Estimate Error Parameter=0 Prob > |T|

1 Intercept 2.73981 0.21287 12.871 0.0000

Linear -0.13417 0.03020 -4.443 0.0000

Quadratic 0.00382 0.00101 3.784 0.0002

2 Intercept 4.63944 0.07436 62.393 0.0000

Linear -0.24436 0.01357 -18.012 0.0000

Quadratic 0.00672 0.00050 13.446 0.0000

3 Intercept 5.40945 0.08307 65.120 0.0000

Linear -0.05071 0.00477 -10.642 0.0000

Sigma 1.07648 0.01412 76.248 0.0000

Group membership

1 (%) 19.13401 3.20149 5.977 0.0000

2 (%) 53.98672 3.07810 17.539 0.0000

3 (%) 26.87927 2.25105 11.941 0.0000

BIC= -5464.63 (N= 3300) BIC= -5455.78 (N= 660) AIC= -5431.07 ll= -5420.07

**Table A9 Suppl. The estimate and standard error for CDAI from the final latent growth curve models in patients with early RA**

Maximum Likelihood Estimates

Model: Censored Normal (CNORM)

Standard T for H0:

Group Parameter Estimate Error Parameter=0 Prob > |T|

1 Intercept 17.47919 5.10688 3.423 0.0006

Linear -4.92036 2.67208 -1.841 0.0657

Quadratic 0.14598 0.08296 1.760 0.0786

2 Intercept 13.43166 0.70783 18.976 0.0000

Linear -1.02603 0.11915 -8.611 0.0000

Quadratic 0.02755 0.00429 6.425 0.0000

3 Intercept 20.09627 1.17282 17.135 0.0000

Linear -0.26857 0.06271 -4.283 0.0000

4 Intercept 34.25551 1.22109 28.053 0.0000

Linear -3.54068 0.20500 -17.272 0.0000

Quadratic 0.10132 0.00745 13.593 0.0000

5 Intercept 30.35779 3.36484 9.022 0.0000

Linear -0.95197 0.33561 -2.837 0.0046

Quadratic 0.03951 0.01404 2.815 0.0049

6 Intercept 50.77133 1.86237 27.262 0.0000

Linear -2.44266 0.34088 -7.166 0.0000

Quadratic 0.03550 0.01296 2.738 0.0062

7 Intercept 39.42606 2.08565 18.903 0.0000

Linear 0.60539 0.40241 1.504 0.1326

Quadratic -0.03750 0.01682 -2.229 0.0258

Sigma 7.79670 0.11498 67.812 0.0000

Group membership

1 (%) 1.92472 1.15653 1.664 0.0962

2 (%) 44.41535 3.53874 12.551 0.0000

3 (%) 22.10099 2.63452 8.389 0.0000

4 (%) 19.52070 2.64172 7.389 0.0000

5 (%) 4.88729 1.77691 2.750 0.0060

6 (%) 4.26467 0.92859 4.593 0.0000

7 (%) 2.88628 0.91988 3.138 0.0017

BIC= -11735.16 (N= 3300) BIC= -11713.43 (N= 660) AIC= -11652.79 ll= -11625.79

**Table A10 Suppl. The estimate and standard error for DAS28-ESR from the final latent growth curve models in patients with established RA**

Model: Censored Normal (CNORM)

Standard T for H0:

Group Parameter Estimate Error Parameter=0 Prob > |T|

1 Intercept 2.27354 0.07339 30.978 0.0000

Linear -0.10421 0.01295 -8.050 0.0000

Quadratic 0.00332 0.00049 6.732 0.0000

2 Intercept 5.04022 0.11777 42.798 0.0000

Linear -0.33144 0.02108 -15.724 0.0000

Quadratic 0.00915 0.00078 11.667 0.0000

3 Intercept 3.25070 0.06393 50.848 0.0000

Linear -0.01955 0.00365 -5.362 0.0000

4 Intercept 4.44399 0.08586 51.760 0.0000

Linear -0.00311 0.00558 -0.557 0.5775

5 Intercept 5.55743 0.12518 44.395 0.0000

Linear -0.11526 0.00881 -13.080 0.0000

6 Intercept 6.90936 0.31238 22.119 0.0000

Linear -0.11228 0.01867 -6.015 0.0000

7 Intercept 5.86058 0.11532 50.818 0.0000

Linear 0.00168 0.00830 0.203 0.8392

Sigma 0.89609 0.00900 99.584 0.0000

Group membership

1 (%) 18.26309 1.50812 12.110 0.0000

2 (%) 14.20290 1.69599 8.374 0.0000

3 (%) 29.79388 1.99716 14.918 0.0000

4 (%) 18.06616 1.83958 9.821 0.0000

5 (%) 10.28172 1.67817 6.127 0.0000

6 (%) 3.25301 1.20252 2.705 0.0068

7 (%) 6.13923 1.20522 5.094 0.0000

BIC= -10019.32 (N= 6300) BIC= -10000.81 (N= 1260) AIC= -9941.71 ll= -9918.71

**Table A11 Suppl. The estimate and standard error for CDAI from the final latent growth curve models in patients with established RA**

Maximum Likelihood Estimates

Model: Censored Normal (CNORM)

Standard T for H0:

Group Parameter Estimate Error Parameter=0 Prob > |T|

1 Intercept 8.78749 0.44543 19.728 0.0000

Linear -0.71408 0.07219 -9.892 0.0000

Quadratic 0.02059 0.00281 7.321 0.0000

2 Intercept 15.82930 0.57271 27.639 0.0000

Linear -0.21131 0.03161 -6.685 0.0000

3 Intercept 23.29061 1.00082 23.272 0.0000

Linear 0.03568 0.06752 0.528 0.5973

4 Intercept 33.56932 1.23124 27.265 0.0000

Linear -3.46523 0.22566 -15.356 0.0000

Quadratic 0.09776 0.00799 12.234 0.0000

5 Intercept 36.50780 1.18029 30.931 0.0000

Linear 0.15673 0.21214 0.739 0.4601

Quadratic -0.01391 0.00867 -1.604 0.1087

6 Intercept 40.82880 1.23850 32.966 0.0000

Linear -1.68371 0.21884 -7.694 0.0000

Quadratic 0.01517 0.00902 1.681 0.0927

7 Intercept 52.48774 2.76345 18.994 0.0000

Linear 0.64905 0.49962 1.299 0.1940

Quadratic -0.04518 0.01990 -2.270 0.0232

Sigma 7.39860 0.07683 96.300 0.0000

Group membership

1 (%) 37.07878 2.39572 15.477 0.0000

2 (%) 31.06627 2.26437 13.720 0.0000

3 (%) 8.89478 1.27828 6.958 0.0000

4 (%) 10.88735 1.59368 6.832 0.0000

5 (%) 4.44137 0.68964 6.440 0.0000

6 (%) 6.98857 1.15978 6.026 0.0000

7 (%) 0.64288 0.24053 2.673 0.0075

BIC= -22031.77 (N= 6300) BIC= -22010.85 (N= 1260) AIC= -21944.04 ll= -21918.04
