# Supplementary material for: Disease activity trajectories for early and established rheumatoid arthritis: Real-world data from a rheumatoid arthritis cohort
Source: PLoS One. 2022 Sep 7;17(9):e0274264. doi: 10.1371/journal.pone.0274264 (PMC9451079; doi:10.1371/journal.pone.0274264)
Supplement: Attachment — Submitted filename: Review Comments to the Authors_responses.docx [file pone.0274264.s002.docx]

**PONE-D-22-02930**

**Disease Activity Trajectories for Early and Established Rheumatoid Arthritis:  Real-World Data from a Rheumatoid Arthritis Cohort**

**PLOS ONE**

**Editor and Reviewer comments:**

**Editors:**

2. Please provide additional details regarding participant consent. In the ethics statement in the Methods and online submission information, please ensure that you have specified what type you obtained (for instance, written or verbal, and if verbal, how it was documented and witnessed). If your study included minors, state whether you obtained consent from parents or guardians. If the need for consent was waived by the ethics committee, please include this information.

Authors’ response: Thanks for this comment. We have moved ethics statement to method section after data collection, which has emphasized all patients, signed informed consent.

3. Thank you for stating the following in the Acknowledgments Section of your manuscript:

“OBRI was funded by peer-reviewed grants from CIHR (Canadian Institute for Health Research), Ontario Ministry of Health and Long-Term Care (MOHLTC), Canadian Arthritis Network (CAN) and unrestricted grants from: Abbvie, Amgen, Aurora, Bristol-Meyers Squibb, Celgene, Gilead, Hospira, Janssen, Lilly, Medexus, Merck, Novartis, Pfizer, Roche, Sandoz, Sanofi, & UCB Acknowledgment: Dr. Bombardier held a Canada Research Chair in Knowledge Transfer for Musculoskeletal Care and a Pfizer Research Chair in Rheumatology”

We note that you have provided additional information within the Acknowledgements Section that is not currently declared in your Funding Statement. Please note that funding information should not appear in the Acknowledgments section or other areas of your manuscript. We will only publish funding information present in the Funding Statement section of the online submission form.

Please remove any funding-related text from the manuscript and let us know how you would like to update your Funding Statement. Currently, your Funding Statement reads as follows:

“The authors received no specific funding for this work.”

Please include your amended statements within your cover letter; we will change the online submission form on your behalf.

Authors’ response: Thanks for this useful comment. We have removed funding statement in the manuscript and added into cover letter. We kindly request you to amend the online submission based on this funding statement.

4. In your Data Availability statement, you have not specified where the minimal data set underlying the results described in your manuscript can be found. PLOS defines a study's minimal data set as the underlying data used to reach the conclusions drawn in the manuscript and any additional data required to replicate the reported study findings in their entirety. All PLOS journals require that the minimal data set be made fully available. For more information about our data policy, please see [http://journals.plos.org/plosone/s/data-availability](https://urldefense.com/v3/__http:/journals.plos.org/plosone/s/data-availability__;!!CjcC7IQ!bZzEpKLCyezCaqssKrF2EW1Rt8OGEyMWFS__ucPWOs7ouxK8RqxfEUTIBB-DUjU9gTiU$).

Upon re-submitting your revised manuscript, please upload your study’s minimal underlying data set as either Supporting Information files or to a stable, public repository and include the relevant URLs, DOIs, or accession numbers within your revised cover letter. For a list of acceptable repositories, please see [http://journals.plos.org/plosone/s/data-availability#loc-recommended-repositories](https://urldefense.com/v3/__http:/journals.plos.org/plosone/s/data-availability*loc-recommended-repositories__;Iw!!CjcC7IQ!bZzEpKLCyezCaqssKrF2EW1Rt8OGEyMWFS__ucPWOs7ouxK8RqxfEUTIBB-DUpIUty3G$). Any potentially identifying patient information must be fully anonymized.

Important: If there are ethical or legal restrictions to sharing your data publicly, please explain these restrictions in detail. Please see our guidelines for more information on what we consider unacceptable restrictions to publicly sharing data: [http://journals.plos.org/plosone/s/data-availability#loc-unacceptable-data-access-restrictions](https://urldefense.com/v3/__http:/journals.plos.org/plosone/s/data-availability*loc-unacceptable-data-access-restrictions__;Iw!!CjcC7IQ!bZzEpKLCyezCaqssKrF2EW1Rt8OGEyMWFS__ucPWOs7ouxK8RqxfEUTIBB-DUrwk1kwO$). Note that it is not acceptable for the authors to be the sole named individuals responsible for ensuring data access.

We will update your Data Availability statement to reflect the information you provide in your cover letter.

Authors’ response: Thanks for this comment.

We are unable to share our data publicly due to ethical and legal restrictions. The data used for this analysis has been collected over the past 12 years. The consent form which participants signed to participate in this study does not give us permission to share the data with third parties. Also, the authors are not the legal owners of this data. The data was collected under the auspices of University Health Network (an academic hospital), therefore the authors are not the custodians of this data. The data is protected by UHN Research Ethics Board and their authorization is required for any use of this data. We have added this explanation in our cover letter.

5. One of the noted authors is a group or consortium “OBRI investigators”. In addition to naming the author group, please list the individual authors and affiliations within this group in the acknowledgments section of your manuscript. Please also indicate clearly a lead author for this group along with a contact email address.

Authors’ response: Thanks for this useful comment. We have added the acknowledgment section at the end of manuscript for all OBRI investigators led by Dr Claire Bombardier. We have provided a list of the investigators with their affiliation as a table in this section.

6. Your ethics statement should only appear in the Methods section of your manuscript. If your ethics statement is written in any section besides the Methods, please move it to the Methods section and delete it from any other section. Please ensure that your ethics statement is included in your manuscript, as the ethics statement entered into the online submission form will not be published alongside your manuscript.

Authors’ response: Thanks for this comment. We moved ethics statement from first page to the method section.

**Reviewers:**

Reviewer #1: The authors analyzed the disease course of early and established RA according to trajectories group identified by LCGM. They found that different measure of disease activity may show different pattern of disease course; specifically lower remission rates in CDAI than in DAS28-ESR over 2-years follow up. The reviewer think that the authors need to add the additional analysis and discussion as listed below.

1. The reviewer could not find the definition of major comorbidities in this study. Please add the definition of major comorbidities in the method section.

Authors’ response: Thanks for this useful comment. We have added the definition for the main comorbidities in the method section.

2. The authors just compared the number of main comorbidities between early RA and established RA groups in table 1. The authors should add the comparisons of each comorbidity based on the detail of the above definition between early RA and established RA groups.

Authors’ response: Thanks for this useful comment. We have added a comparison of the main comorbidities in early versus established RA in the table 1.

3. Because the authors found the significant difference about remission rates based on DAS28-ESR or CDAI in an identical early RA cohort, the authors should discuss which is better to use DAS28-ESR or to use CDAI in clinical practice to prevent radiographic progression or to achieve better outcome of patients’ quality of life.

Authors’ response: Thanks for this comment. As we have not looked at radiographic progression and quality of life in this manuscript, comparison of these outcomes between these measures are not straightforward and therefore we preferred not to comment on these.

4. In table 3, please add the comparisons of each comorbidity based on the detail of the above definition.

Authors’ response: Thanks for this useful comment. We have added a comparison of the main comorbidities in the table 3.

5. In the results section, please add the analysis for CDAI trajectories group characteristics in patients with early RA.

Authors’ response: Thanks for this comment. We think a comparison of baseline characteristics between seven CDAI trajectories in early RA would not be useful and informative because of small numbers in each cell.

6. In the results section, please add the analysis for DAS28-ESR trajectories group characteristics in patients with established RA.

Authors’ response: Thanks for this comment. We think a comparison of baseline characteristics between seven DAS28-ESR trajectories in established RA would not be useful and informative because of small numbers in each cell.

7. In the results section, please add the analysis for CDAI trajectories group characteristics in patients with established RA.

Authors’ response: Thanks for this comment. We think a comparison of baseline characteristics between seven CDAI trajectories in established RA would not be useful and informative because of small numbers in each cell.

8. Please add the discussion about selection bias as one of the limitation of the study because a significant proportion of patients were excluded at the final analysis as shown in Figure 1.

Authors’ response: Thanks for this useful comment. We have added selection bias as a limitation for generalizability of results in the discussion.

Reviewer #2: I have a number of comments regarding this paper
1. Literature review: There are a number of papers that have performed similar analyses to the one in this paper which have not been cited. For example, Dagliati et al, A&R (2020); RA-MAP Consortium, Ther Adv in MusculSkelet Dis (2021); RA-MAP Consortium, RMD Open, 2018; McWilliams et al, AR&T (2016). These should be added. It would also be useful for the authors to explain what they work will add to that which is out there already.

Authors’ response: Thanks for comments. We have discussed these papers in the discussion. McWilliams et al 2016 did not use composite DAS28 or CDAI as outcomes for disease course and comparison of their results with our finding was not directly possible, therefore, we did not discuss this article in our manuscript.

2. Definition of LDA, MD, HD, Remission: The authors should provide in the main text of the paper the definitions for LDA, MD, HD, Remission with respect to DAS28-ESR or CDAI, rather than relegating only to the footnotes under Figures 2b, 3a
and 3b.

Authors’ response: Thanks for this useful comment. We have added definitions in the method section.

3. Validation: There is an opportunity here to perform some form of validation of the clustering/subgroup structures found using DAS28-ESR and CDAI across early and established RA either for example through some form of cross-validation or by splitting the data into a testing and training set. My concern is that the clusters/subgroups found are not necessarily robust either across similar populations or even within the same population, especially the very small clusters/subgroups. These subgroups would be more meaningful/convincing if the authors could show at the least that they are stable or they associate meaningfully to later health outcomes such as damage progression (through use of X-rays) or functional disability.

Authors’ response: Thanks for this comment. We believe that cross-tabulation of seven subgroups of CDAI and DAS28 ESR, because of small numbers in each cell, would not be very informative or useful.

4. Given that the number and presumably the clusters themselves may be different depending on whether DAS28-ESR or CDAI is used, it would be helpful to present the cross-tabulations of the clustering structure based on DAS28-ESR and the clustering structure based on CDAI for both early and established RA, so as to understand how these structure differ. Also it would be helpful for the authors to discuss what the implications of these differing clustering structures on treat-to-target strategies which aim to improve health outcomes of patients with RA.

Authors’ response: Thanks for this comment. We are not sure that how informative and useful e cross-tabulation of seven subgroups of CDAI and DAS-28 ESR because of small numbers in each cell.

5. Statistical Methods: The authors should provide more details on how they assign patients to subgroups based on the latent growth curve model (LCGM or LGCM?).

Authors’ response: Thanks for this useful comment. We have added more details about LCGM in data analysis section.

6. In Figures 2a, 2b, 3a and 3b , the authors should make the figures clearer. For example, (i) explicitly state that the solid lines correspond to the observed and the dashed lines to the expected; (ii) add a legend indicating that Group 1 corresponds to the red lines, Group 2 to the green lines and Group 3 to the blue lines, and make it clear that the numbers alongside the lines are the percentage of patients within these groups; and (iv) add the confidence bands around the curves to reflect the uncertainty.

Authors’ response: Thanks for this comment. In each figure, the legend shows the number and related color (please see Figure 2a). To address the reviewer’s comments we have added a footnote indicating that the solid line refers to the observed and the dashed line to the fitted values. We added confidence intervals for figure 2a. We believe adding confidence bands for figure 2b, 3a and 3b with seven subgroups makes the figures very busy and hard to read. However, we added confidence bands for the mean in related tables in the text and supplementary section.

7. The actual results (estimates and standard errors) from the final latent growth curve models should be provided in the Supplementary.

Authors’ response: Thanks for this useful comment. We have added estimates and errors for final models for DAS28-ESR and CDAI in early and established RA in the supplementary section.

8. The authors need to discuss the possible impact selection bias has on their findings given that they only look at patients with complete outcome data (DAS28-ESR and CDAI) at baseline and all follow-up visits up to 24-months.

Authors’ response: Thanks for this useful comment. We have added selection bias as a limitation for generalizability of results in the discussion.

Reviewer #3: Dear authors
Thank you for an interesting paper that attempts to identify the trajectories of disease activity in a large cohort of patients with rheumatoid arthritis. I think that the paper reads well.
I have some concerns regarding the methods and presentation of data.

Major concerns:
1. I find it odd that all patients with early RA can be categorised into just three categories of DAS28. Were there no patients who remained in high disease activity for the duration of the two years? Similarly, did no patients go from MD to HD in the established RA group? To this end it would be useful to include either standard deviations or IQR in table 2.

Authors’ response: Thanks for this useful comment. We have added 95% CI for mean reported in table 2.

2. I missed tables similar to table 2 and 3 for the other categorisations? If there is not enough space in the manuscript, then included them in the appendix.

Authors’ response: Thanks for this useful comment. We have added mean (95% CI) of DAS28-ESR for established RA and CDAI for early and established RA as tables A2-A4 suppl .

3. Were there some patients who participated in both early and late

Authors’ response: No

4. Although telephone calls are made every 6-months, CDAI and DAS28 cannot be collected by telephone. How often were the routine care visits and is it possible that routine care visits were initiated by patients who experienced high disease activity, thus giving a false impression of persistently high disease activity?

Authors’ response: Thanks for this useful comment. We have followed patients from their enrolment in the registry regardless of their disease status. However, as the reviewer mentioned patients with high disease activity are more likely to visit their physician. We have added this as some potential limitation in the discussion.

Minor
1.The abstract states that "Only 14.2% of established RA reached DAS28-ESR remission" whereas the discussion states "Using DAS28-ESR we found that 48% of established patients were in remission and low disease status at
enrolment and retained their disease status over two years’ follow-up. Almost 27.8% of patients
experienced some degree of improvement". While both may be true I think there should be a better alignment between the message given in the abstract and in the discussion.

Authors’ response: Thanks for this comment. To avoid confusion between the abstract and discussion we deleted the statement in the discussion starting with “ 48% with remission and LDA at enrolment…”

2. What was the average number of visits in each group? It would be helpful if you could show the number of patients with visit at each time point in each trajectory to help the reader judge the validity of the information.

Authors’ response: Thanks for this comment. The average number of visits was 13 (SD: 6.6) for early RA and 14 (SD: 7.0) for established RA. We added this information in the result section.
