# Supplementary material for: Disease activity trajectories for early and established rheumatoid arthritis: Real-world data from a rheumatoid arthritis cohort
Source: PLoS One. 2022 Sep 7;17(9):e0274264. doi: 10.1371/journal.pone.0274264 (PMC9451079; doi:10.1371/journal.pone.0274264)
Supplement: Attachment — Submitted filename: Review Comments to the Authors_responses_round 2_20220808.docx [file pone.0274264.s003.docx]

6. Review Comments to the Author

Please use the space provided to explain your answers to the questions above. You may also include additional comments for the author, including concerns about dual publication, research ethics, or publication ethics. (Please upload your review as an attachment if it exceeds 20,000 characters)

Reviewer #1: (No Response)

Reviewer #2: 1. The authors have not addressed (or attempted to address) my concern regarding validation (Comment 3). As mentioned in my earlier review, "My concern is that the clusters/subgroups found are not necessarily robust either across similar populations or even within the same population, especially the very small clusters/subgroups. These subgroups would be more meaningful/convincing if the authors could show at the least that they are stable or they associate meaningfully to later health outcomes such as damage progression (through use of X-rays) or functional disability."
Authors’ response: Thanks for this comment. As recommended, we addressed stability by performing cross-tabulation of CDAI and DAS28 ESR in both early and established RA and added results in the supplementary material (Tables A3 and A6 Suppl). As an exploratory analysis, to show some meaningful association between DAS28 subgroups and functional disability (HAQ-DI and pain) we have added a table for early RA patients, in the supplementary material (Table A7 Suppl). We also added a related statement in the discussion.

2.Related to my Comment 4 in my previous review, I believe it is unsatisfactory to not provide the cross-tabulations of the clustering structures of DAS28-ESR by CDAI for early RA and established RA, irrespective of whether some of the cells have small numbers. These cross-tabulations without much effort can be provided as supplementary material. Additionally the authors have not discussed what the implications of these differing clustering structures on treat-to-target strategies which aim to improve health outcomes of patients with RA.

Authors’ response: Thanks for this comment. We did cross-tabulation of CDAI and DAS28 ESR in both early and established RA and added them in the supplementary material (Tables A3 and A6 Suppl). We also discussed the association between subgroups identified by CDAI and DAS28 and implication of CDAI and DAS28 as two most common disease activity indices in the clinic setting. We have added a paragraph on treat-to target strategies in the discussion.

3. In the Discussion, the authors added the following sentence "There is also a possibility of more clinic visits by patients with high disease activity compared to those with LDA status, which may affect the impression of disease course toward persistent high disease activity in these patients." This does not make sense to me as all patients analysed had the same number of visits (i.e. 5). The authors should clarify.

Authors’ response: Thanks for this comment. To clarify this, all patients had data at baseline, 6 month, 12 months, and 24 months follow-up. However, they may have had different numbers of visits over 24 months (Mean: 5.7; SD: 2.0).

4. In the Data Analysis section, the authors need to add to the sentence "Subjects are then assigned to the group they most likely belong", the criterion (e.g. based on that group being estimated to have the highest posterior probability of the subject being allocated to it) that is used to make this allocation.
Authors’ response: Thanks for this comment. We added this to the sentence.

5. Table A1 Suppl: The column headings (Group1, Group2, Group3, Group4) do not make sense.

Authors’ response: Thanks for this comment. We removed them.

6. Table A2 Suppl: Based on the 24-month mean value of 12.8 for Group 6 (VHD-LDA), it is unclear why this group is described as VHD-LDA, when LDA based on CDAI is defined to have a CDAI value <= 10.

Authors’ response: Thanks for this comment. We corrected them.

Reviewer #3: Thank you for revising this interesting manuscript according to our suggestions. I have no further comments.
